# Effect of Lipid Raft Disruptors on Cell Membrane Fluidity Studied by Fluorescence Spectroscopy

**DOI:** 10.3390/ijms232213729

**Published:** 2022-11-08

**Authors:** Ádám Horváth, János Erostyák, Éva Szőke

**Affiliations:** 1 Department of Pharmacology and Pharmacotherapy, Medical School, University of Pécs, Szigeti Str. 12, H-7624 Pécs, Hungary; 2 National Laboratory for Drug Research and Development, Magyar Tudósok Krt. 2, H-1117 Budapest, Hungary; 3 Department of Pharmacology, Faculty of Pharmacy, University of Pécs, Rókus Str. 2, H-7624 Pécs, Hungary; 4 János Szentágothai Research Centre and Centre for Neuroscience, University of Pécs, Ifjúság Str. 20, H-7624 Pécs, Hungary; 5 Department of Experimental Physics, Faculty of Sciences, University of Pécs, Ifjúság Str. 6, H-7624 Pécs, Hungary

**Keywords:** cholesterol, fluorescens spectroscopy, LAURDAN, lipid raft, methyl-beta-cyclodextrin, myriocin, sphingomyelinase

## Abstract

Lipid rafts are specialized microdomains in cell membranes, rich in cholesterol and sphingolipids, and play an integrative role in several physiological and pathophysiological processes. The integrity of rafts can be disrupted via their cholesterol content—with methyl-β-cyclodextrin (MCD) or with our own carboxamido-steroid compound (C1)—or via their sphingolipid content—with sphingomyelinase (SMase) or with myriocin (Myr). We previously proved by the fluorescent spectroscopy method with LAURDAN that treatment with lipid raft disruptors led to a change in cell membrane polarity. In this study, we focused on the alteration of parameters describing membrane fluidity, such as generalized polarization (*GP*), characteristic time of the GP values change—Center of Gravity (*τ*_CoG_)—and rotational mobility (*τ*_rot_) of LAURDAN molecules. Myr caused a blue shift of the LAURDAN spectrum (higher GP value), while other agents lowered GP values (red shift). MCD decreased the CoG values, while other compounds increased it, so MCD lowered membrane stiffness. In the case of *τ*_rot,_ only Myr lowered the rotation of LAURDAN, while the other compounds increased the speed of *τ*_rot_, which indicated a more disordered membrane structure. Overall, MCD appeared to increase the fluidity of the membranes, while treatment with the other compounds resulted in decreased fluidity and increased stiffness of the membranes.

## 1. Introduction

The field of biological and pharmacological research has been impacted by the lipid raft hypothesis since it was first introduced by Karnovsky [1]. Lipid rafts are specialized microdomains in the plasma membrane, which are rich in cholesterol, sphingolipids, and gangliosides [2]. The distribution of the raft components is different in the inner and outer leaflets of the raft regions. Sphingomyelins and glycophospholipids are present in the outer leaflet, while the inner leaflet is rich in glycerophospholipids. Cholesterol, as the main component, shows equal distribution in the rafts [2,3,4]. Not only the structure of the rafts is special, but also the function. Lipid rafts play an essential role in several physiological and pathophysiological processes, such as in signal transduction pathways connected to the immune, cardiovascular, and nervous systems [5,6,7], as well as in tumor formation [7,8,9,10]. It is clearly known that several ion channels are associated with lipid rafts; for instance, the GABA (gamma-aminobutyric acid) receptor, AMPA (2-amino-3-(5-methyl-3-oxo-1,2-oxazol-4-yl)propanoic-acid) glutamate receptor, nicotinic acetylcholine receptor, and some Transient Receptor Potential (TRP) receptors—which are in the main interest of our research group [11,12,13,14,15,16,17,18]. Therefore, investigation of the integrity of rafts can be a useful technique to follow receptor functions.

The integrity of the rafts can be disrupted in two different ways, one is depletion of the different constituents, such as cholesterol or sphingomyelins, and the other is inhibition of the synthesis of the components. To deplete cholesterol from the rafts, methyl-beta-cyclodextrin (MCD) is the commonly used agent, which forms a complex with cholesterol [19]. The sphingomyelin content can be depleted by sphingomyelinase (SMase), which is a natural enzyme that hydrolyzes the sphingomyelins to ceramide and phosphocholine [20]. To inhibit the formation of rafts by blocking the synthesis of sphingolipids, myriocin (Myr) is used, which is a serine-palmitoyl-transferase inhibitor and this enzyme catalyzes the rate-limit step of the sphingolipid synthesis [21].

Our group previously described that the lipid raft disruption by MCD, SMase, Myr, and a novel carboxamido-steroid compound (C1), which depletes cholesterol from the membrane, could lead to the inhibition of the TRP Vanilloid 1 (TRPV1) and TRP Ankyrin 1 (TRPA1) receptors in different in vitro and in vivo conditions [16,17,18,22,23,24]. Despite the in vitro and in vivo data, the exact molecular mechanism of the inhibition by lipid raft disruption is still unknown. To somehow clarify this issue, we used earlier the fluorescent spectroscopy method to characterize native Chinese Hamster Ovary (CHO) cells after incubation with the lipid raft disruptors [17,18,22]-For fluorescent spectroscopy 6-dodecanoyl-N,N-dimethyl-naphthylamine (LAURDAN) is a commonly used polarity sensitive dye, which is suitable to investigate model membrane, such as POPC-LUV (2-Oleoyl-1-palmitoyl-sn-glycero-3-phosphocholine large unilamellar vesicle), DOPC-LUV (1,2-dioleoyl-sn-glycero-3-phosphocholine large unilamellar vesicle), DOPC-GUV (1,2-dioleoyl-sn-glycero-3-phosphocholine giant unilamellar vesicle), and DPPC-GUV (Dipalmitoylphosphatidylcholine giant unilamellar vesicle), as well as membranes of living cells [23,24,25,26,27]. Originally LAURDAN was introduced by Parasassi and co-workers in membrane biophysics [28,29]. LAURDAN is a polarity-sensitive probe that can incorporate into the phospholipid bilayer of membranes—both inner and plasma membrane—[23,30,31], and its excitation and emission properties depend on the actual hydration state of the membrane [23]. Different methods were developed in the last decades to use LAURDAN as a membrane-sensitive probe to determine membrane polarity and fluidity. Fluorescent spectroscopy is the less complicated one, and fluorescent microscopy and two-photon microscopy are the more complex methods [32].

Based on our earlier steady-state measurements [17,18,24], we aimed in this study to investigate the effect of SMase, Myr, MCD, and C1 treatment on membrane fluidity in native CHO cells. Fluorescence spectroscopy delivers several parameters which could be used well to describe how the microenvironment around LAURDAN changes. Using more parameters supports a more complex analysis revealing different details of the membrane structure. A methodology based on complex fluorescence spectroscopy analysis gives us a versatile tool for describing competing changes in cell membranes.

## 2. Results

### 2.1. Interpretation

The interpretation of the detailed parameters below can be individual and/or collective. Individually, they characterize the behavior of LAURDAN molecules in the membrane and, taken together, provide information on the microviscosity of the membrane. It should be noted, however, that fluorescence spectroscopy is not suitable for the selective analysis of the plasma membrane since the inner and outer membrane systems cannot be separated, but LAURDAN molecules are present in both membrane systems [31]. For this reason, the results were evaluated qualitatively, and relative conclusions were drawn from the direction of change. It is also notable that our results are presented with intrinsic errors, which represent the uncertainties of the experimental method.

### 2.2. Fluorescence Decays

**Figure 1:**
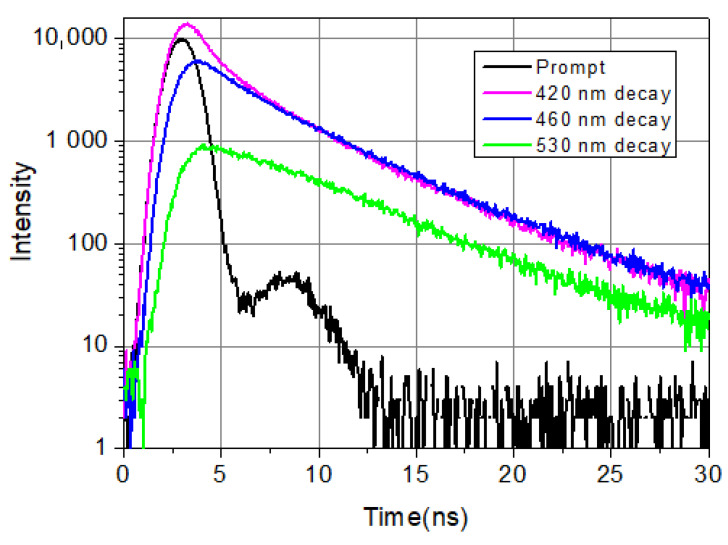
LAURDAN emission decays at 420 nm (violet), 460 nm (blue), and 530 nm (green). LAURDAN in cell suspension, treated with Smase. *λ*_ex_ = 369 nm.

Figure 1 shows typical decays from the blue side, the center, and the red side of the LAURDAN’s emission. Among the four exponential components, there is a very fast one, faster than the resolution limit of the Nanolog spectrofluorometer. Its value is taken as 0.01 ns; the exact value is not known. Nevertheless, it is important to include this fast component in the deconvolution procedure; this way, all the other decay components and their pre-exponentials can be calculated precisely. The possible reasons for the ultrafast decay component might be scattering of excitation light not cut totally by the emission monochromator and color cut-off filter, fast component of solvent relaxation, etc.

It can be seen that on the shorter wavelength (*λ* = 420 nm), the fluorescence decays faster compared to the longer wavelength (*λ* = 530 nm) emission in the first few nanoseconds. This is a consequence of excited state processes. At longer times (*t* > 10 ns), the speed of decaying is the same at every wavelength, which indicates that emission arrives from relaxed molecules. These decays show the complexity of the temporal evolution of LAURDAN’s non-monoexponentially decaying emission. Details of exited state relaxation are discussed below.

### 2.3. Characteristic Time of Center-of-Gravity (τ_CoG_)

**Figure 2:**
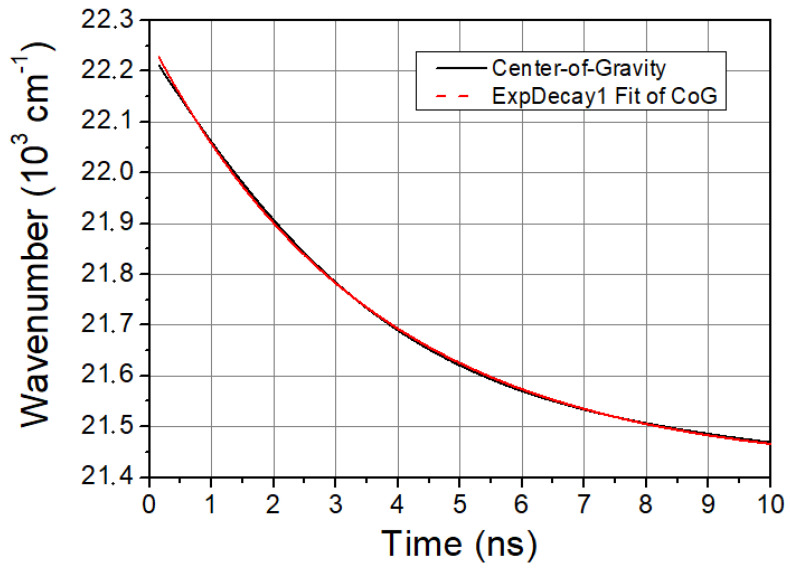
CoG function of LAURDAN. Black line is the CoG function of LAURDAN and red dashed line is a mono-exponential fit giving excellent cover with the measured CoG function.

The characteristic time of solvent relaxation can be determined from the temporal evolution of the spectral shift of LAURDAN’s emission. A faster spectral shift corresponds to a faster kinetics of the solvent relaxation process. The speed of solvent relaxation is directly related to the rotational mobility of the water molecules around the LAURDAN within the membrane, and this is often referred to as membrane viscosity. If the τCoG is low, that means a faster rotational motion of water molecules around LAURDAN and lower membrane viscosity, while if the value is high, it means a slower rotational motion of water molecules around LAURDAN, higher membrane viscosity, and stiffness. The time evolution of CoG function is monoexponential in all cases studied. A typical graph of it is shown in Figure 2.

The calculated values of the characteristic times of the CoG function are presented in Table 1.

For SMase, Myr, and C1, there is an increase in τCoG values compared to non-treated samples, which characterizes a slowing of the spectral shift, suggesting some increase in membrane stiffness. In contrast, for MCD, a significant decrease in τCoG is seen, which clearly implies faster solvent relaxation, i.e., reduced stiffness.

### 2.4. Generalized Polarization (GP)

For the determination of the GP, the intensity values obtained by integrating the fluorescence decay from treated and control (non-treated) samples are used, comparing the results obtained in each case with the self-control. A higher GP value means a more restricted motion of LAURDAN in the membrane, while lower values are referred to as a less restricted motion of LAURDAN in the membrane. The calculated *GP* values are summarized in Table 2.

In the case of SMase, MCD, and C1, the *GP* values decreased after the treatment, indicating a red shift in the spectral pattern. The red shift is clear evidence of a transition from the liquid-ordered to the liquid-disordered phase, i.e., the substances alter the microenvironment of the membranes to a greater or lesser extent. In the case of Myr, there is a higher *GP* value, which means a blue shift in the spectral pattern, and this indicates a transition of the membrane into a more ordered phase.

### 2.5. Time-Resolved Area-Normalized Spectra (TRANES)

**Figure 3:**
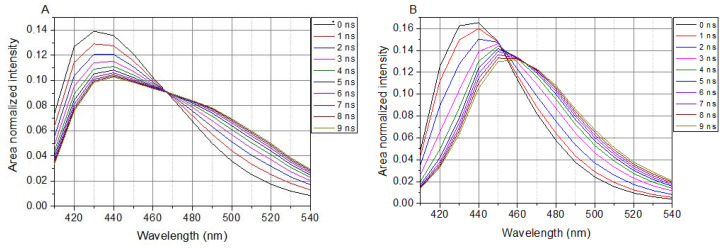
TRANES of LAURDAN. (**A**), Laurdan in cell suspension; (**B**), Laurdan in non-cell solution. *λ*_ex_ = 369 nm.

In cell-containing solutions, LAURDAN’s TRANES show an isoemissive point (*λ* = 466 nm in Figure 3A), which is clear evidence of a two-state reversible process within the excited states of LAURDAN. This indicates the existence of locally excited (LE) and charge transfer (CT) states. Any way of treatment with the lipid raft disruptors, there is no influence on the presence of the isoemissive point.

The presence of SMase, Myr, MCD, or C1 does not change the appearance of the isoemissive point; these compounds have an effect on the temporal evolution of processes around LAURDAN in the membrane.

In a buffer solution (without cells), LAURDAN’s TRANES does not show an isoemissive point, but rather a continuous shift of emission spectrum can be observed (Figure 3B), which corresponds to a continuous relaxation.

### 2.6. Time-Resolved Anisotropy

**Figure 4:**
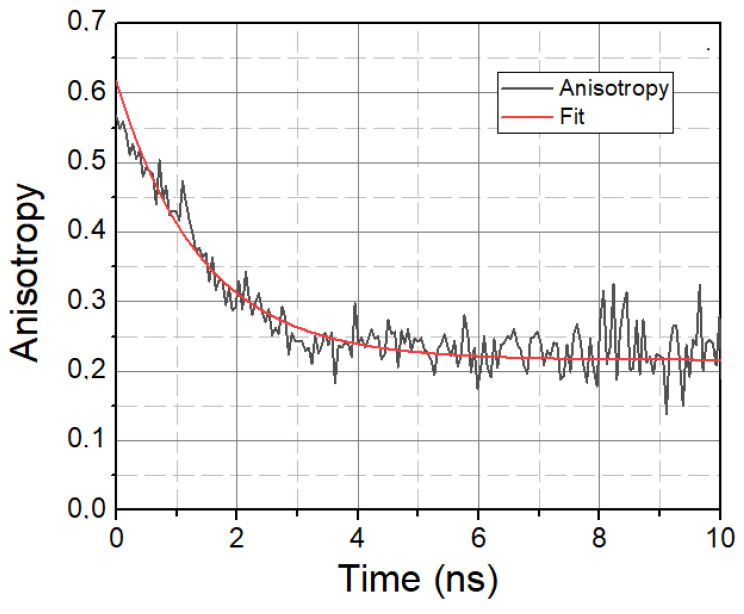
Anisotropy decay of LAURDAN. *λ* = 450 nm. Black—Anisotropy data. Red—1-exponential fit.

In addition to solvent relaxation, we also investigated the mobility property of LAURDAN molecules, the characteristic parameter of which is the rotational correlation time *τ*_rot_, calculated using time-resolved anisotropy decays. A typical anisotropy decay is displayed in Figure 4.

A higher *τ*_rot_ value means the slower rotational motion of LAURDAN, while a lower *τ*_rot_ value means the faster rotational motion of LAURDAN. The calculated rotational lifetime values based on treated and untreated samples are given in Table 3.

The variation in *τ*_rot_ gives information about the rotational properties of LAURDAN in the membrane, i.e., how much the molecule is confined in the membrane. For SMase, MCD, and C1, the values decrease compared to the control, implying faster rotation and, thus, less confinement of LAURDAN molecules. In contrast, for Myr, there is an increase, implying slower rotation and, thus, more confined LAURDAN molecules in the membrane.

## 3. Discussion

The manipulation of lipid rafts has opened up an alternative method in pharmacological studies to investigate both the microenvironment of membranes and the function of receptor proteins located within them. An experimental tool for the environment and fluidity of membranes is the LAURDAN fluorescent dye, whose fluorescence parameters can be used to infer the current hydration level of the membrane, i.e., the ratio of liquid-ordered to liquid-disordered phases [23,24,33]. In addition, the characteristic parameters of fluorescence and anisotropy decays can be used to characterize fluidity [34,35,36]. Our research group has previously demonstrated that the cholesterol-depletor MCD and C1 alter cell membrane polarity by lowering GP values in steady-state conditions [17,18]. We also described that SMase did not change the membrane polarity, while Myr caused a significant elevation in fluorescence intensity, which meant a shift to the more liquid-ordered phase [22].

**Figure 5:**
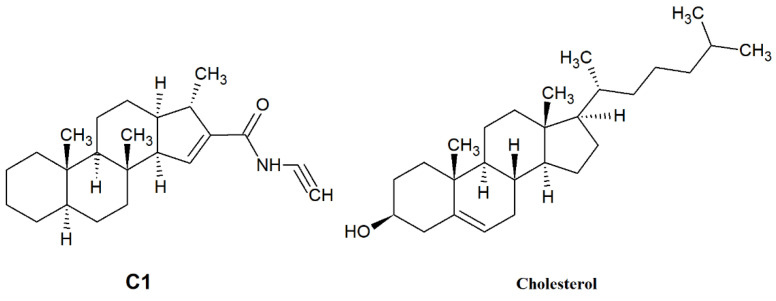
Structure of C1 compound and cholesterol.

In our current study, we investigated the effect of SMase, Myr, MCD, and C1 on cell membrane fluidity with fluorescence spectroscopy using LAURDAN. First, we proved with the TRANES measurement—the existence of the isoemissive point—that LAURDAN molecules are incorporated into the membrane; however, it is important that with fluorescence spectroscopy, the cell membrane and inner membrane system cannot be distinguished. The change in *GP* values for SMase, MCD, and C1 showed a red shift, indicating that the membrane has changed to a more disordered direction. These results are in agreement with previous findings from other groups. Weber and colleagues investigated the effect of temperature changes and cholesterol depletion (by MCD) or supplementation (MCD:cholesterol complex) and found that increased temperature or MCD treatment decreased membrane stiffness, while cholesterol enrichment showed the opposite effect [37]. Furthermore, Startek and co-workers investigated the effect of MCD on mouse TRPA1 localization in raft region by Total Internal Reflection Fluorescence Microscopy and gradient separation methods. They found a high co-localization of mouse TRPA1 and flotillin-2 (a lipid raft marker), which is significantly decreased after MCD treatment, indicating an alteration in the membrane structure [38]. In the case of Myr treatment, the GP change showed a different shift from the others in the blue direction, in agreement with our previously described result [22], suggesting that the cell membrane has moved to a more ordered phase. This observation is in contrast to a recently reported result where two-photon microscopy was used to investigate the effects of decreased sphingolipid supply as well as Myr treatment [39,40]. Due to the impairment of sphingolipid synthesis and decreased sphingolipid supply, LAURDAN fluorescence analysis showed a significant decrease in membrane rigidity, which was also confirmed with breakthrough force measurements [39]. Furthermore, a significant decrease in LAURDAN GP was observed after 5 min of 2.5 µM Myr treatment, but the authors noted that other lipidomic analyses had measured elevated glycerophospholipid levels, which compensated to some extent for the effect of Myr [40,41]. The difference in results was presumably due to the difference in concentration (100 nM vs. 2.5 µM), the duration of treatment (24 h vs. 5 min), and the difference in method since both measurements were performed on CHO cells. The two-photon microscopy allowed selective examination of the plasma membrane, whereas fluorescence spectroscopy provided information on the whole membrane system of the cell. From the rate of change of *CoG* values over time, we can infer the relaxation of the solvent surrounding LAURDAN, which is related to membrane stiffness and, thus, indirectly to membrane microviscosity. The decrease of *τ*_CoG_ values in MCD-treated samples is a clear sign of faster solvent relaxation, indicating lower stiffness. However, in the case of SMase, Myr, and C1, there was a smaller to larger increase in *τ*_CoG_ values as a result of treatment, indicating a slowing of the spectral shift, i.e., increased stiffness. In the case of SMase, our observation may be explained by the fact that phosphocholine and ceramide synthesized during hydrolysis of sphingomyelins, remaining in the membrane, slow down solvent relaxation processes [20,42]. In the case of Myr, the processes involved in glycerophospholipid formation mentioned above may be responsible [40,41,43], while in the case of C1, a structural similarity to cholesterol (Figure 5) may explain the slowing of solvent relaxation and spectral shift, as it may substitute cholesterol in the membrane.

For *τ*_rot_, we found that SMase, MCD, and C1 treatment resulted in reduced values compared to controls, indicating a less confined and, therefore, faster spinning LAURDAN molecule, that is, a lower microviscosity. In the case of Myr, we also obtained the opposite result, indicating a more confined presence of LAURDAN. The explanation for decreased *τ*_rot_ value after SMase treatment could be the structural differences between sphingomyelins and the products of SMase digestion, phosphocholine and ceramide. In the case of MCD and C1, the depletion of cholesterol provides a clear explanation for the faster rotation of LAURDAN. In the case of Myr, the elevated compensatory formation of glycerophospholipids, which can somehow counter the inhibitory effect of Myr, may explain the increased *τ*_rot_ values, which indicates that the LAURDAN molecules have slower rotational properties.

## 4. Materials and Methods

### 4.1. Drugs and Chemicals

C1 was synthesized by our collaborators at the University of Pannonia, Department of Organic Chemistry. SMase, MCD, Myr, and LAURDAN were purchased from Sigma (St. Louis, MO, USA). C1 and LAURDAN were dissolved in dimethyl sulfoxide (DMSO) to obtain a 10 mM stock solution, and Myr was also dissolved in DMSO to obtain a 5 mM stock. MCD was freshly dissolved in an extracellular solution (ECS), and SMase—which was in a glycerol-buffered solution—was also freshly diluted with ECS to the final concentration. ECS parameters were the following: 160 mM NaCl, 2,5 mM KCl, 1 mM CaCl_2_, 2 mM MgCl_2_, 10 mM HEPES, 10 mM glucose, pH 7,3. For the treatment, we used the listed concentrations: 30 mU (SMase), 100 nM (Myr), 100 µM (C1), 10 mM MCD, and 40 µM LAURDAN, respectively.

### 4.2. Sample Preparation

Native CHO cells were placed on 24-well plates, and the cells were treated with the lipid raft disruptors in ECS at 37 °C in a humidified atmosphere with 5% CO_2_. The concentrations and the treatment durations were the following: 30 mU SMase, 100 µM C1, 10 mM MCD for 45 min, and 100 nM Myr for 24 h, respectively. Cells were then washed 3 times with phosphate-buffered saline (PBS) and incubated with 40 µM LAURDAN solution in ECS for 40 min at the same condition. Cells were then washed 3 times with PBS, scraped from the plates, and transferred into 1 mL PBS. For the fluorescent measurement, two samples were prepared simultaneously, and before the measurements, the samples were pooled. For every single treatment, a self-control (non-treated) sample was used. Treated and control samples originating from the same preparation were measured within a few hours. This ensures proper background for observation of the effects of disruptors. Due to the different cell numbers in our samples, the control samples were different from day to day, and therefore the comparison can be made between the coupled control and treated samples.

### 4.3. Spectrofluorometer Setup

To record time-resolved fluorescence decays and anisotropy decays, a HORIBA Jobin-Yvon Nanolog FL3-2iHR spectrofluorometer equipped with NanoLEDs as excitation sources were used. The wavelength of NanoLED pulses was 369 nm, which fits very well with LAURDAN’s excitation spectrum. The typical temporal pulse width was 1.0 ns. Built-in deconvolution software of Nanolog was used to analyze fluorescence and anisotropy decays. On the emission side, in front of the entrance slit of the emission monochromator, a long pass glass filter transparent over 399 nm was applied to reduce the flux of the excitation photons scattered from the samples. Samples were measured in a 4 mm path length quartz cuvette (Hellma 104F-QS), and their temperature was set and controlled by a Thermo Scientific circulating bath AC200-A25 to 20 °C.

### 4.4. Determination of Membrane Fluidity Parameters

To study the membrane fluidity, both fluorescence decays and anisotropy decays were measured. In this section, we describe how parameters characterizing the membrane fluidity was determined from the raw measured data.

#### 4.4.1. Fluorescence Decays, Time-Emission Matrices

Fluorescence decay curves were measured through the whole emission range of LAURDAN (400–540 nm, 14 decays, Δ*λ* = 10 nm). In a deconvolution procedure, they were fitted by the 4-exponential function; this gave a satisfying fit at all wavelengths. It was necessary because—over the intrinsic non-exponential decay nature of LAURDAN—there was a strong spectral shift of emission in time, which appeared as complexity of decay. The lifetime components were only proper fitting parameters describing precisely enough this complex decay shape; they were not attributed to any separate emission species. The non-exponential fluorescence decay of LAURDAN at several wavelengths can be described very well using the calculated fitting parameters. With these determined decay parameters, the original fluorescence decay curves of LAURDAN can be reproduced within 0.03%.

From the series of decay curves, Time-Emission Matrices (TEM) were constructed. In the vertical direction of TEM (Time) there are decay curves. In the horizontal direction of TEM (Emission wavelength) there are time-resolved emission spectra that could be constructed from the intensity data of the consecutive decay curves at the same time point.

#### 4.4.2. Characteristic Time of CoG

The speed of solvent relaxation can be described by the time evolution of the Center-of-Gravity [*CoG*(*t*)] function of the time-resolved emission spectra [44]. *CoG*(*t*) can be calculated with the following equation (Equation (1)):

(1) $$CoG\left(t\right)=\frac{\sum I\left(\upsilon ,t\right)\upsilon }{\sum I\left(\upsilon ,t\right)}$$

It is important to mention that the fluorescence decays were collected in wavelength scale; thus the following transformation (Equation (2)) is needed for the precise calculation:

(2) $$CoG\left(t\right)=\frac{\sum I\left(\lambda ,t\right)\lambda^{-1}}{\sum I\left(\lambda ,t\right)}$$

In our calculations, data from all of the measured decays were used (14 decays, 410–540 nm).

A higher value of *CoG*(*t*) means faster conformational change of LAURDAN followed by solvent relaxation of neighboring water molecules. This is a consequence of higher membrane stiffness.

#### 4.4.3. Time-Resolved Area-Normalized Spectra

Time-resolved spectra show the spectral shapes of the emission of LAURDAN at different time points. The temporal evolution of the shape and spectral position of these spectra reflects the temporal change of energetic distance of the excited and ground states involved in the emission. Time-Resolved Area-Normalized Spectra (TRANES) are special versions of time-resolved spectra and could be used well in spectral analysis [*I*_AN_(*λ*,*t*)] [44]. TRANES were constructed from the time-resolved spectra of TEM as their area under the spectra were normalized to the same value. Equation (3) gives the mathematical formula for producing TRANES:

(3) $$I_{\mathrm{AN}}\left(\lambda ,t\right)=\frac{S\left(0\right)}{S\left(t\right)}I\left(\lambda ,t\right)$$

where

(4) $$S\left(t\right)=\sum_{\lambda_{\min}}^{\lambda_{\max}}I\left(\lambda ,t\right)$$

If the spectra cross each other at the same point, we have a so-called isoemissive point, which is clear evidence of a two-state reversible process within the excited states of the molecule studied. The lack of the isoemissive point indicates a continuous shift in the excited state rather than a two-state reaction.

#### 4.4.4. Generalized Polarization

To quantify the spectral changes, Generalized Polarization function (*GP*) is widely used [23,24,33], too. From emission data, we calculated the emission *GP* function with Equation (5):

(5) $$GP=\frac{I_{430}-I_{500}}{I_{430}+I_{500}}$$

where *I*_430_ and *I*_500_ stand for the intensities in fluorescence emission spectrum at 430 nm and 500 nm, respectively. These steady-state intensity data were calculated as integrals of fluorescence decays of LAURDAN with Equation (6):

(6) $$I=\int_{0}^{\infty }I\left(t\right)dt$$

The samples were handled for roughly 3 h as fresh; thus, there was not enough time to measure both time-resolved and steady-state data one after another from the same sample. That is why steady-state intensity data were calculated rather than measured.

The higher value of *GP* reflects a blue-shifted spectrum, which is an indication of a more restricted motion of LAURDAN in the membrane. The lower value of *GP* reflects a red-shifted spectrum, and it is an indication of less restricted motion of LAURDAN in the membrane.

Please note that the name “Generalized Polarization” has no connection to any optical polarization. This quantity is widely used in articles and describes spectral changes. Its name just remembers that its definition is analogous to the definition of physical quantity “polarization”; there is just a mathematical similarity of definitions.

#### 4.4.5. Rotational Correlation Time

Anisotropy decay *r*(*t*) was calculated as follows:

(7) $$r\left(t\right)=\frac{I_{VV}\left(t\right)-GI_{VH}\left(t\right)}{I_{VV}\left(t\right)+2GI_{VH}\left(t\right)}$$

where *G* = *I_HV_/I_HH_* is a correction factor [41]. *I_VV_*(*t*) and *I_VH_*(*t*) are the emission decays measured in the presence of horizontal and vertical polarizers on excitation and emission sides.

*τ*_rot_ was determined from the time-resolved anisotropy decay measurements made at the spectral maximum of the emission (450 nm). It provides information on the mobility of LAURDAN molecules in the phospholipid bilayer, i.e., their confinement in the membrane [35,36].

In the time-resolved anisotropy measurements *τ*_rot_ was determined using the following formula (Equation (8)):

(8) $$r\left(t\right)=\left(r_{0}-r_{\infty }\right)\exp\left(-\frac{t}{\tau_{\mathrm{rot}}}\right)+r_{\infty }$$

where *r*(*t*) is the calculated anisotropy decay. This expression can even be applied at hindered rotors when the anisotropy does not decay to zero.

A higher value of *τ*_rot_ means slower rotation of LAURDAN, which is clear evidence for increased microviscosity around it within the membrane.

#### 4.4.6. Steady-State Spectra

**Figure 6:**
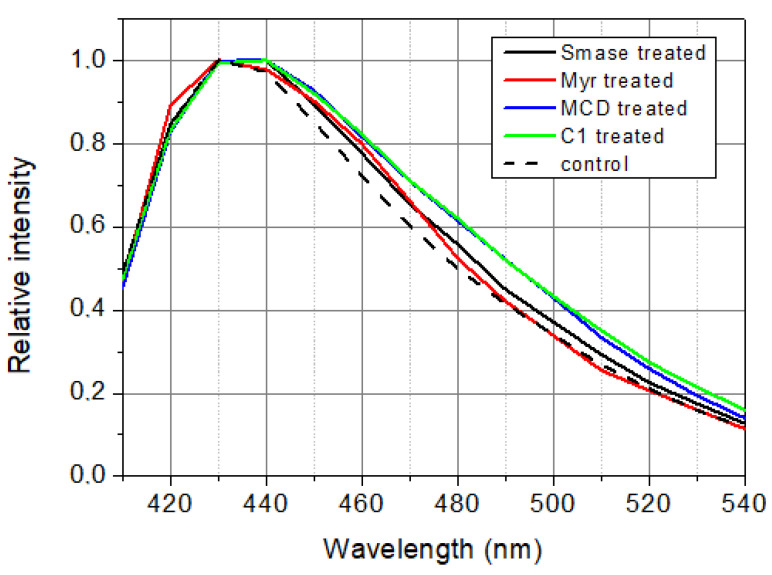
Calculated steady-state spectra of LAURDAN. *λ*_ex_ = 369 nm.

Samples studied were used in fluorescence measurements for not more than three hours after preparation because of the degradation of cells. Under this time window, both treated and control samples had to be measured. It means that there was time only for time-resolved measurements but not for steady-state measurements. That is why steady-state spectra (Figure 6) have been calculated from lifetime and pre-exponential decay parameters as time integral of the decay at a certain wavelength:

(9) $$I\left(\lambda \right)=\overset{\infty }{\underset{0}{\int }}I\left(\lambda ,t\right)dt=\sum a_{n}\tau_{n}$$

where *a_n_* and *τ_n_* are the pre-exponential and the lifetime for the *n*-th decay component.

## 5. Conclusions

Based on our previous fluorescent spectroscopy result [17,18,22] and the current data, we conclude that treatment with lipid raft disruptors—SMase, Myr, MCD, C1—can modify membrane fluidity and stiffness and therefore, this effect can lead to modification of different membrane and membrane-related protein functions, such as the inhibition of TRPV1 and TRPA1 activation.

## Figures and Tables

**Table 1:** τ_CoG_ values determining the rate of change of CoG in control and treated samples. Errors are the intrinsic measurement errors.

Sample Name	Control *τ*_CoG_ (ns)	Treated *τ*_CoG_ (ns)	Direction of Change
30 mU SMase	3.93 ± 0.12	4.17 ± 0.13	Increase
100 nM Myr	3.32 ± 0.10	3.53 ± 0.11	Increase
10 mM MCD	3.37 ± 0.10	3.07 ± 0.09	Decrease
100 µM C1	4.64 ± 0.14	5.45 ± 0.16	Increase

**Table 2:** *GP* values in control (non-treated) and treated samples. Errors are the intrinsic measurement errors.

Sample Name	Control *GP*	Treated *GP*	Direction of Change
30 mU SMase	0.49 ± 0.01	0.46 ± 0.01	more Disordered
100 nM Myr	0.46 ± 0.01	0.49 ± 0.01	more Ordered
10 mM MCD	0.45 ± 0.01	0.40 ± 0.01	more Disordered
100 µM C1	0.54 ± 0.02	0.39 ± 0.01	more Disordered

**Table 3:** *τ*_rot_ values in control and treated samples. Errors are the intrinsic measurement errors.

Sample Name	Control *τ*_rot_ (ns)	Treated *τ*_rot_ (ns)	Direction of Change
30 mU SMase	1.60 ± 0.05	1.40 ± 0.04	Faster
100 nM Myr	1.45 ± 0.04	1.69 ± 0.05	Slower
10 mM MCD	1.60 ± 0.05	1.55 ± 0.05	Faster
100 µM C1	1.71 ± 0.05	1.56 ± 0.05	Faster

## Data Availability

The original data presented in the study are included in the article. Further inquiries can be directed to the corresponding author.

## Abbreviations

AMPA	2-amino-3-(5-methyl-3-oxo-1,2-oxazol-4-yl)propanoic-acid
C1	carboxamido-steroid compound
CHO	Chinese Hamster Ovary
CT	charge transfer
DMSO	dimethyl sulfoxide
DOPC	1,2-dioleoyl-sn-glycero-3-phosphocholine
DPPC	Dipalmitoylphosphatidylcholine
ECS	extracellular solution
GABA	Gamma-aminobutyric-acid
GP	generalized polarization
GUV	giant unilamellar vesicles
LAURDAN	6-dodecanoyl-N,N-dimethyl-naphthylamine
LE	locally excited
LUV	large unilamellar vesicles
MCD	methyl-beta-cyclodextrin
Myr	myriocin
PBS	phosphate-buffered saline
POPC	2-Oleoyl-1-palmitoyl-sn-glycero-3-phosphocholine
SMase	sphingomyelinase
TEM	Time Emission Matrix
TRANES	Time-Resolved Are-Normalized Spectra
TRP	Transient Receptor Potential
TRPA1	Transient Receptor Potential Ankyrin 1
TRPV1	Transient Receptor Potential Vanilloid 1
τrot	rotational correlation time
τCoG	characteristic time of Center of Gravity
